# West Nile Virus in Brazil

**DOI:** 10.3390/pathogens10070896

**Published:** 2021-07-15

**Authors:** Érica Azevedo Costa, Marta Giovanetti, Lilian Silva Catenacci, Vagner Fonseca, Flávia Figueira Aburjaile, Flávia L. L. Chalhoub, Joilson Xavier, Felipe Campos de Melo Iani, Marcelo Adriano da Cunha e Silva Vieira, Danielle Freitas Henriques, Daniele Barbosa de Almeida Medeiros, Maria Isabel Maldonado Coelho Guedes, Beatriz Senra Álvares da Silva Santos, Aila Solimar Gonçalves Silva, Renata de Pino Albuquerque Maranhão, Nieli Rodrigues da Costa Faria, Renata Farinelli de Siqueira, Tulio de Oliveira, Karina Ribeiro Leite Jardim Cavalcante, Noely Fabiana Oliveira de Moura, Alessandro Pecego Martins Romano, Carlos F. Campelo de Albuquerque, Lauro César Soares Feitosa, José Joffre Martins Bayeux, Raffaella Bertoni Cavalcanti Teixeira, Osmaikon Lisboa Lobato, Silvokleio da Costa Silva, Ana Maria Bispo de Filippis, Rivaldo Venâncio da Cunha, José Lourenço, Luiz Carlos Junior Alcantara

**Affiliations:** 1 Departamento de Medicina Veterinária Preventiva, Universidade Federal de Minas Gerais, Belo Horizonte 31270-901, Brazil; azevedoec@yahoo.com.br (É.A.C.); mariaisabel.guedes@gmail.com (M.I.M.C.G.); beatrizsenra.santos@gmail.com (B.S.Á.d.S.S.); ailavet@yahoo.com.br (A.S.G.S.); 2 Laboratório de Flavivírus, Instituto Oswaldo Cruz, Fundação Oswaldo Cruz, Rio de Janeiro 21040-360, Brazil; giovanetti.marta@gmail.com (M.G.); flaviallevy@yahoo.com.br (F.L.L.C.); nielircf@gmail.com (N.R.d.C.F.); ana.bispo@ioc.fiocruz.br (A.M.B.d.F.); 3 Laboratório de Genética Celular e Molecular, Universidade Federal de Minas Gerais, Belo Horizonte 31270-901, Brazil; vagner.fonseca@gmail.com (V.F.); faburjaile@gmail.com (F.F.A.); joilsonxavier@live.com (J.X.); 4 Departamento De Morfofisiologia Veterinária, Universidade Federal do Piauí, Teresina 64049-550, Brazil; catenacci@ufpi.edu.br; 5 KwaZulu-Natal Research Innovation and Sequencing Platform (KRISP), School of Laboratory Medicine and Medical Sciences, College of Health Sciences, University of KwaZulu-Natal, Durban 4041, South Africa; tulioDNA@gmail.com; 6 Coordenação Geral dos Laboratórios de Saúde Pública/Secretaria de Vigilância em Saúde, Ministério da Saúde (CGLAB/SVS-MS), Brasília 70719-040, Brazil; 7 Laboratório Central de Saúde Pública, Fundação Ezequiel Dias, Belo Horizonte 30510-010, Brazil; felipeemrede@gmail.com; 8 Diretoria de Vigilância em Saúde, Fundação Municipal de Saúde, Teresina 64600-000, Brazil; marceloadrianoneuro@gmail.com; 9 Seção de Arbovirologia e Febres Hemorrágicas, Instituto Evandro Chagas, Ministério da Saúde, Ananindeua 70058-900, Brazil; dannifh@hotmail.com (D.F.H.); danielemedeiros@iec.pa.gov.br (D.B.d.A.M.); 10 Setor de Clínica de Equinos, Hospital Veterinário, Campus Pampulha, Universidade Federal de Minas Gerais Escola de Veterinária, Belo Horizonte 31270-901, Brazil; rpamaranhao@yahoo.com; 11 Department of Large Animal Clinic, Universidade Federal de Santa Maria, Rio Grande do Sul 97105-900, Brazil; renata.farinelli@ufsm.br; 12 Coordenacao Geral das Arboviroses, Secretaria de Vigilância em Saúde/Ministério da Saúde, Brasília 70058-900, Brazil; karina.cavalcante@saude.gov.br (K.R.L.J.C.); noely.moura@saude.gov.br (N.F.O.d.M.); alessandro.romano@saude.gov.br (A.P.M.R.); 13 Organização Pan-Americana da Saúde, Organização Mundial da Saúde, Brasília 40010-010, Brazil; meloc@paho.org; 14 Centro de Ciências Agrárias, Departamento de Clínica e Cirurgia Veterinária, Universidade Federal do Piauí, Teresina 64049-550, Brazil; jackvet08@hotmail.com; 15 Faculdade de Ciências da Saúde, Medicina Veterinária, Urbanova, São José Dos Campos, UNIVAP-Universidade Vale do Paraíba, São Paulo 12245-720, Brazil; jjveterinario@hotmail.com; 16 Departamento de Clínica e Cirurgia Veterinárias, Escola de Veterinária, Universidade Federal de Minas Gerais, Belo Horizonte 31270-901, Brazil; teixeiraraffa@gmail.com; 17 Laboratório de Genética e Conservação de Germoplasma, Campus Prof. Cinobelina Elvas, Universidade Federal do Piauí, Bom Jesus, Piauí 64049-550, Brazil; osmaikonlobato@gmail.com (O.L.L.); silvokleio@ufpi.edu.br (S.d.C.S.); 18 Coordenacao dos Laboratorios de Referencia, Oswaldo Cruz Foundation, Rio de Janeiro 21040-360, Brazil; rivaldo.cunha@fiocruz.br; 19 Department of Zoology, University of Oxford, Oxford OX1 3PS, UK; jose.lourenco@zoo.ox.ac.uk

**Keywords:** West Nile virus, genomic monitoring, molecular detection, Brazil

## Abstract

*Background:* West Nile virus (WNV) was first sequenced in Brazil in 2019, when it was isolated from a horse in the Espírito Santo state. Despite multiple studies reporting serological evidence suggestive of past circulation since 2004, WNV remains a low priority for surveillance and public health, such that much is still unknown about its genomic diversity, evolution, and transmission in the country. *Methods:* A combination of diagnostic assays, nanopore sequencing, phylogenetic inference, and epidemiological modeling are here used to provide a holistic overview of what is known about WNV in Brazil. *Results:* We report new genetic evidence of WNV circulation in southern (Minas Gerais, São Paulo) and northeastern (Piauí) states isolated from equine red blood cells. A novel, climate-informed theoretical perspective of the potential transmission of WNV across the country highlights the state of Piauí as particularly relevant for WNV epidemiology in Brazil, although it does not reject possible circulation in other states. *Conclusion:* Our output demonstrates the scarceness of existing data, and that although there is sufficient evidence for the circulation and persistence of the virus, much is still unknown on its local evolution, epidemiology, and activity. We advocate for a shift to active surveillance, to ensure adequate preparedness for future epidemics with spill-over potential to humans.

## 1. Introduction

West Nile virus (WNV), a member of the *Flaviviridae* family, was first identified in the West Nile district of Uganda in 1937, but nowadays, it is commonly found in Africa, Europe, North America, the Middle East, and Asia [1,2,3]. WNV transmission is maintained in a mosquito–bird cycle, for which the genus *Culex*, in particular *Cx. pipiens* and *quinquefasciatus*, are considered the principal vectors [4]. WNV can infect humans, equines, and other mammals, but these are considered “dead-end” hosts, given their weak potential to function as amplifying hosts to spread infection onwards [5,6]. Around 80% of WNV infections in humans are asymptomatic, while the rest may develop mild or severe disease. Mild disease includes fever, headache, tiredness, and vomiting [7,8], while severe disease (neuroinvasive) is characterized by high fever, coma, convulsions, and paralysis [7,8]. Equine infections can occasionally cause neurological disease and death [7,8], such that equines typically serve as sentinel species for WNV outbreaks with potential for spill-over into human populations.

Genome detection of WNV in South America was originally reported in horses (Argentina in 2006) and captive flamingos (Colombia, in 2012) [9,10]. The first ever sequenced genome in Brazil was in 2018, when the virus was isolated from a horse with severe neurological disease in the Espírito Santo state [11]. Despite multiple studies reporting serological evidence suggestive of past WNV circulation in Brazil (e.g., [11,12,13]) and reports of human WNV disease in confirmed cases in the Piauí state [13], much is unknown about genomic diversity, evolution, and transmission dynamics across the country. The reality of WNV in Brazil is likely characterised by endemic circulation within the mosquito–bird cycle [14,15,16,17], with occasional transmission to humans. The so far lack of reported human epidemics with significant public health impact remains a puzzle, given that Brazil harbors the necessary vectors, avian species, and climate—combination amenable at sustaining endemicity [18]. Several factors potentially contribute to the seemingly silent circulation of WNV in the country [19], such as the lack of surveillance interest and resources, rates of mild human WNV disease, co-circulation of other mosquito-borne viruses that cause similar clinical spectrums, and diagnostics and screening of animals and humans well past the time of infection, which critically hampers viral detection and confirmation.

In this study, we aim at providing a holistic perspective of what is known about WNV circulation in Brazil. In addition to previously reported evidence of WNV circulation, we also report new genetic evidence of WNV circulation in three Brazilian states. We further provide a climate-informed, theoretical assessment of the transmission potential of WNV across Brazil, revealing spatio-temporal patterns of interest. The lack of surveillance data hampers more in-depth analyses and therefore obscures our current understanding of WNV epidemiology, evolution, and transmission in the country. Recently, some European countries have witnessed a shift from a similar surveillance and epidemiological situation to that of Brazil, to observing recurrent WNV epidemics with spill-over to human populations [18,19,20,21]. We argue that active surveillance initiatives are necessary in Brazil in the near future to ensure preparedness of future WNV epidemics with public health impact.

## 2. Results

### 2.1. Novel Evidence of WNV Circulation in Three Brazilian States

Samples (RBCs) from three horses with suspected WNV infection obtained from southern (Minas Gerais and São Paulo) and northeastern (Piauí) Brazilian states were sent for molecular diagnosis at the Departamento de Medicina Veterinária Preventiva at the Federal University of Minas Gerais (UFMG).

**Figure 1:**
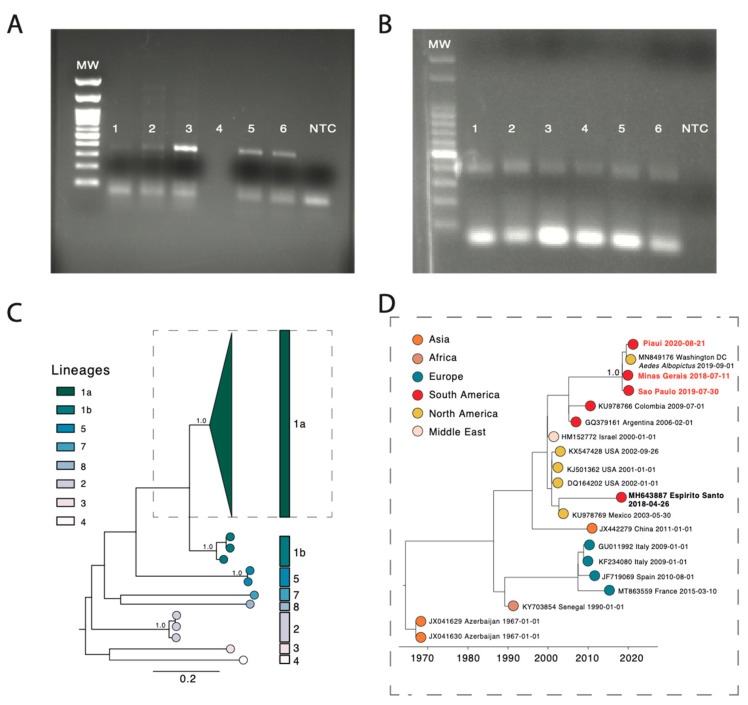
Investigation of WNV infections in Brazil, between July 2018 and September 2020, and estimated transmission potential. (**A**,**B**) Agarose gel electrophoresis of amplicons from assay for WNV. (**A**) nested RT-PCR. MW (Molecular weight ladder), 100 bp DNA Ladder RTU, Kasvi; 1—plasma of horse from São Paulo; 2—buffy coat of horse from São Paulo; 3—washed RBC of horse from São Paulo; 4—blank negative control using during the nested RT-PCR; 5 and 6—positive control (synthetic gene); NTC, no template control (using since the extraction); expected amplicon size: 370 bp. (**B**) Multiplex PCR. MW (Molecular weight ladder), Fluorescent 100 bp DNA Ladder, Cellco, Jena Bioscience; 1—horse form Minas Gerais (pair primers); 2—horse form Minas Gerais (odd primers); 3—horse form Sao Paulo (pair primers); 4—horse form Sao Paulo (impair primers); 5—horse form Piaui (pair primers); 6—horse form Piaui (odd primers); NTC, no template control (using since the extraction); expected amplicon size: 400 bp. (**C**) Midpoint rooted maximum-likelihood phylogeny of WNV genomes, showing major lineages. The scale bar is in units of substitutions per site (*s/s*). Support for branching structure is shown by bootstrap values at nodes. (**D**) Time-resolved maximum likelihood tree showing the WNV strains belonged to the 1a lineage. Colors indicate geographic location of sampling. The new Brazilian WNV strains are shown with text in red.

RNAs were extracted from red blood cells and tested using an in-house PCR assay (see Methods section for details). WNV-specific RT-PCR amplification products were obtained by nested PCR (Figure 1A,B), and positive samples were subjected to a newly designer multiplex PCR scheme (Supplementary Table S1) to generate complete genomes sequences by means of portable nanopore sequencing.

Three blood fractions (plasma, buffy coat, and washed RBC) from the horses sampled in São Paulo and Minas Gerais states have been submitted to nested RT-PCR; horse samples from Piaui have been tested only using RBC, which was the only blood fraction available. Diagnostic investigation of alphavirus was also performed using a generic RT-PCR targeting the NSP1 gene, according to [22], in the three blood fractions, with negative results.

The published WNV genome from Brazil (MH643887) was used to generate (mean) 98.4% consensus sequences that formed the target for primer design. The new genomes were deposited in GenBank with accession numbers MW420987, MW420988, and MW420989 (Table 1).

We constructed phylogenetic trees to explore the relationship of the sequenced genomes to those sampled elsewhere globally. We retrieved 2321 WNV genome sequences with associated lineage date and country of collection from GenBank, from which we generated a subset that included the highly supported (>0.9) clade containing the newly WNV strains obtained in this study plus 29 sequences (randomly sampled) from all lineages and performed phylogenetic analysis. An automated online phylogenetic tool to identify and classify WNV sequences was developed (available at: http://krisp.ukzn.ac.za/app/typingtool/wnv/job/9b40f631-51c4-419c-9edf-2206e7cd8d9c/interactive-tree/phylo-WNV.xml accessed on 31 December 2019).

Phylogenies estimated by the newly developed WNV typing tool, along with maximum likelihood methods (Supplementary Figure S1C), consistently placed the Brazilian genomes in a single clade within the 1a lineage with maximum statistical support (bootstrap = 100%) (Supplementary Figure S1).

Time-resolved maximum likelihood tree appeared to be consistent with previous estimates [11] and showed that the new genomes clustered with strong bootstrap support (97%) with a WNV strain isolated from an *Aedes albopictus* mosquito in Washington DC, USA in 2019 (Figure 1D). Interestingly, the new isolates did not group with the previously sequenced genome in 2019 from the Espirito Santo state, suggesting that inter-continental introduction events might be frequent in Brazil.

### 2.2. A Data-Driven WNV Theoretical Perspective

**Figure 2:**
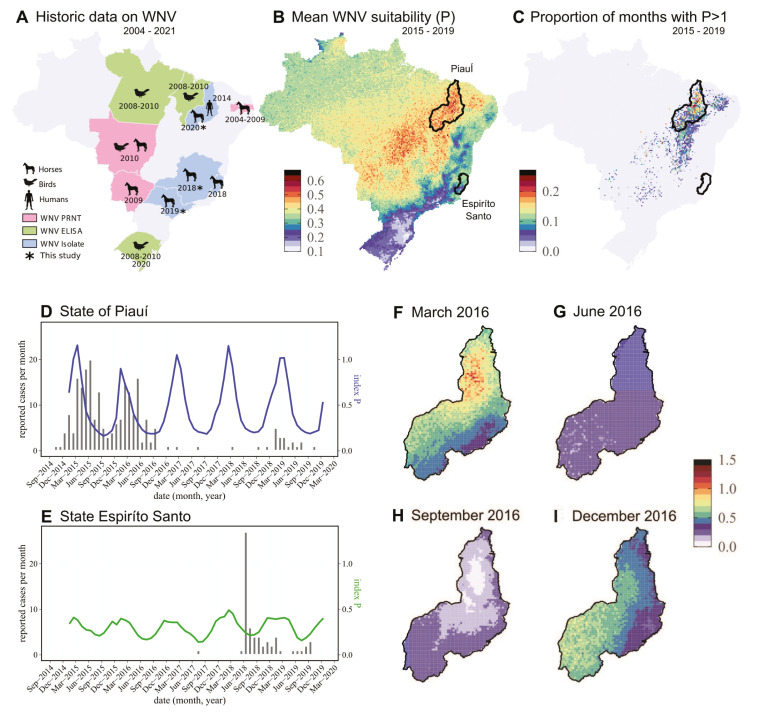
Data-driven epidemiological perspective of WNV in Brazil. (**A**) Mapping of historic evidence for WNV circulation in Brazil, for which the color and symbol legend on the bottom left of the panel define the animal source and methodology. Data are based on a literature review up to 2019 [24], in addition with recently published reports in 2020–2021 [24] and the new data generated in this study. (**B**) Mean estimated transmission potential of WNV (index P) over the period 2015–2019. The color scale on the bottom left of the panel shows the range of the presented values. The black borders mark the boundaries of the Piauí and Espiríto Santo states. (**C**) Proportion of months for which the transmission potential of WNV (index P) was above the value 1, over the period 2015–2019. The color scale on the bottom left of the panel shows the range of the presented values. The black borders mark the boundaries of the Piauí and Espiríto Santo states. (**D**) Time series of suspected reported West Nile fever cases (bars) and estimated transmission potential of WNV (index P, blue line) for the Piauí state. Index P is the average per month, across all data points within the boundaries of the state. (**E**) Time series of suspected reported West Nile fever cases (bars) and estimated transmission potential of WNV (index P, green line) for the Espiríto Santo state. Index P is the average per month, across all data points within the boundaries of the state. (**F**) Spatial snapshot of estimated transmission potential of WNV (index P) for the month of March 2016. Color scale on the right shows the range of the presented values. (**G**) Same as F but for June 2016. (**H**) Same as F but for September 2016. (**I**) Same as F but for December 2016.

We first summarized the past evidence of WNV circulation in Brazil from avian species, equines, and humans, which was achieved via various literature reports using different confirmation methods (Figure 2A) [23]. The first evidence of WNV infection was documented in 2004 in horses in northeastern Brazil (Paraiba state). Since then, serological evidence of WNV infection continued to be documented between 2008 and 2010 and again in 2020 in horses and birds from the southern [24], midwestern (Pantanal), and northern Brazilian regions. In 2014, the first WNV infection in a human was confirmed in the Piauí State (northeast region). In 2018, the first isolation of WNV in Brazil was documented in the Espirito Santo state (southeastern Brazil) when the virus was isolated from the central nervous system (CNS) of a dead horse with neurological manifestations [11]. To these data, we here add the report of the new genetic evidence of WNV circulation in equines occurring between 2018 and 2020, in southern (Minas Gerais, São Paulo) and northeastern (Piauí) states. To the best of our knowledge, it is the first time that evidence of WNV circulation is reported for the states of Minas Gerais and São Paulo.

Using data collected from the Brazilian “Sistema de Informação de Agravos de Notificação” (SINAN) (see Methods and Supplementary Table S2) reported with identifier A923 (“Febre do Nilo”), we explored the current spatio-temporal distribution of suspected cases of West Nile fever. Given the unspecific and unconfirmed nature of these reported cases, we complemented such information with theoretical projections of the spatio-temporal transmission potential of WNV in Brazil. For this, we used a climate-driven suitability measure (index P) previously successfully applied to WNV in the contexts of Israel [25] and Portugal [26] (see Methods).

We mapped the mean index P across Brazil for the period 2015–2019 (Figure 2B) and found estimated transmission potential to be highest in the center of the country along a diagonal latitude–longitude axis crossing from the center–west to the north–east. The regions of the south of the country, similarly to estimations for other mosquito-borne viruses [27], presented the least transmission potential. To assess potential hotspots of (at least temporary) high transmission potential, we calculated the proportion of months (2015–2019) in which the index P was above 1; this particular threshold representing the point above which each female mosquitoes would be theoretically able to infect more than one host during their lifetime. This approach identified regions of Piauí, Bahia, Ceará, Rio Grande do Norte, and Paraíba states as presenting significantly longer periods of time with high index P. In particular, the state of Piauí was captured in its entirety within this estimated spatial hotspot of transmission potential (Figure 2C).

From all states for which there were reported cases, we filtered those that had more than one case per any month during the entire period of 2015–2019, selecting only two states with clear epidemic waves of reported cases: Piauí and Espírito Santo. Coincidently with the results of Figure 2B,C, the state of Piauí reported the largest number of cases in the entire dataset. Using the geographical boundaries of each state, we averaged the index P per month (Figure 2D,E). The resulting time series of transmission potential showed that potential was higher in Piauí compared to Espírito Santo in accordance with the spatial output in Figure 2B,C. It also presented a clear seasonal signal, with peaks occurring on average in February in Piauí (month average = 2.2, summer) and April in Espiríto Santo (month average = 4, autumn). The correlation between reported cases and the index for Piauí was positive (Pearson’s 0.36, Figure 2D), but it was negative for Espiríto Santo (Pearson’s −0.31, Figure 2E). Similar to what has been reported for suitability indices applied to other viruses [27], there was a clear lag between the index and cases for Piauí, with cases lagging behind the index (Figure 2D). Accordingly, shifting the index by one month into the future resulted in a high positive correlation with cases (Pearson’s 0.84).

Finally, to get a grasp of the possible spatio-temporal dynamics of WNV transmission in Piauí, we looked at estimated transmission potential for one of the years with more reported suspected cases (2016) both in space and time (with snapshots at months of March, June, September, and December) (Figure 2F–I). The spatio-temporal snapshots showed that transmission potential was the lowest during winter months, but we also highlighted that this was almost uniform across the state (Figure 2G,H). In contrast, throughout the year, this output highlighted a possible wave of seasonal transmission. This wave would typically start in the southwest just before summer (Figure 2I) and would move to the northeast in the summer (Figure 2F).

## 3. Discussion

Our analyses indicate that additional data are required to better identify routes of WNV importation into and within Brazil and to more generally understand the local transmission dynamics of the virus. Interestingly, our data suggest that the circulation of the virus may have resulted from multiple independent introductions, since the new isolates did not group with the previously sequenced genome in 2019 from the Espirito Santo state. This suggests that intra-continental introduction events due to the mobility of infected birds or mosquitoes might be a more plausible mechanism for the multiple introductions of WNV in South American countries, including Brazil. This scenario is consistent with previous studies that showed that multiple independent introductions into Latin America occurred during the initial outbreak in US in 1999; detailed revision is provided in [28]. While migrating birds are a convenient explanation of WNV dispersal, other possible ways of dispersion exist, such as infected mosquitoes that are accidentally transported via airplane or by road transport [29]. Another likely scenario is commercial legal or ilegal human transportation of birds and/or mosquitoes, which could be transported on airplanes [29].

The current data scarceness prevents definite conclusions on key aspects of WNV epidemiology. For example, given the unconfirmed nature of the reported cases by SINAN for Piauí and Espírito Santo, it is unclear what the proportion of cases truly reflect WNV occurrence and seasonality, hampering our ability to ascertain how representative our theoretical projections are. For Piauí, we would speculate that reported cases may indeed reflect some aspects of WNV seasonality, given that this state had the largest number of cases reported while also being the region of Brazil for which we estimated higher transmission potential and that our estimated transmission potential was well correlated with reported cases (albeit with a possible lag of one month typical of mosquito-borne viruses). At the same time, while inferred trees including the new genome sequences suggest that inter-continental introduction events might be frequent in Brazil, the lack of higher spatio-temporal sampling restricts our ability for definite conclusions on viral movement and persistence.

The phylogenetic and epidemiologic perspectives presented in this study, based on both existing and novel data as well as theoretical projections, suggest that both scenarios of sporadic and endemic local transmission are possible [30]. Similarly to sudden changes in WNV epidemiology and transmission as recently observed in other countries, the occurrence of a WNV outbreak affecting humans in Brazil may simply be a matter of time. Shifting from passive to active WNV screening and sequencing in animal reservoirs (e.g., equines, birds, vectors) in Brazil must be implemented to better understand the virus’ local epidemiology and to be able to act accordingly in preventing and controlling any future epidemics with spill-over to humans.

## 4. Materials and Methods

### 4.1. Sample Collection, Viral RNA Isolation and PCR Screening

Samples (red blood cells, RBCs) from three horses with suspected WNV infection obtained from southern (Minas Gerais and São Paulo) and northeastern (Piauí) Brazilian states were sent for molecular diagnosis at the Laboratório de Patologia Molecular at the Federal University of Minas Gerais (UFMG).

Sample 1 from 11 July 2018 was collected from a 9-month-old female horse in a farm in the state of Minas Gerais, Mangueiras neighbourhood (Sabará), 15 km from the capital Belo Horizonte. Clinical findings were consistent with bilateral blindness. Neurological examination revealed no other abnormalities. The ophthalmological exams (direct and indirect pupillary light reflex (PLR), fluorescein eye stain test, fundus examination, and intraocular pressure) were consistent with retinal disease, mainly with chorioretinitis.

Sample 2 from 30 July 2019 was collected from a 13-year-old male horse that presented seizure episodes, muscle stiffness, tremor retinal, and flaccid paralysis in a farm located in São Bernardo do Campo countryside of the São Paulo state. Twenty-four days after the onset of neurological signs, the animal had severe pain in the forelimbs from laminitis, and it was euthanized due to hoof decumulation.

Sample 3 from 21 August 2020 was collected from a male horse, 5 years old, which died 72 h after presenting neurological signs, in a farm located in the municipality of Parnaíba, Piauí state. The animal presented motor incoordination, paddling movements, loss of sensitivity over the spine column, and behavioral changes. In this municipality, the tenth human case in Brazil was also detected, presenting neuroinvasive disease compatible with WNV infection, confirmed by serological assay (IgM) in both serum and cerebrospinal fluid (CSF) samples during acute and convalescent phases.

Whole blood samples obtained from the three horses were centrifuged at 1260× *g* for 20 min, and the plasma and buffy coat fractions were collected and stored at 4 °C. Red blood cells (RBC) were washed by centrifugation three times in phosphate-buffered saline (PBS) at 1260× *g* for 10 min and stored also at 4 °C [15]. RNA from each unit (washed RBC, plasma and buffy coat) were extracted using the QIAmp Viral RNA Mini kit (Qiagen, Hilden, Germany), following manufacturer’s recommendations.

Diagnostic investigation of arboviruses was performed by a generic RT-PCR targeting the flavivirus non-structural protein 5 (NS5) gene [31] and alphavirus non-structural protein 1 gene (nsP1) [32]. West Nile virus-specific degenerated primers: forward primers (+) AACCKCCAGAAGGAGTSAAR and reverse primers (−) AGCYTCRAACTCCAGRAAGC were used in second reaction of nested PCR targeting the NS5 gene after a genus specific flavivirus RT-PCR amplification [22]. A synthetic gene fragment of partial NS5 gene (gblocks gene fragment, Integrated DNA Technologies) was used as a positive control. The 25 μL PCR “master-mix” comprised 2.5 μL of 10× PCR buffer, 1.5 mM MgCl2, 0.4 μM of each primer (forward and reverse), 0.8 μM dNTP mixture (Phoneutria, Sao Paulo, Brazil), 1 U Taq DNA polymerase (Platinum Taq DNA polymerase; Invitrogen, Carlsbad, CA, USA), 2 μL of template DNA (sample or gBlock), and DNA/RNAse-free water. The thermocycling conditions involved 40 cycles, and reaction conditions were previously reported in [18]. As an internal control for amplification efficiency, primers for the beta actin gene were used. As a negative control for the reactions, we used RNA extracted from equine washed RBC, plasma, and buffy coat that previously tested negative for arboviruses, equine herpesvirus 1 and 4, and borna disease. The amplicons were analyzed by 1% (*w/v*) agarose gel electrophoresis, stained with ethidium bromide, and visualized under UV light. Nested PCR were performed for equine herpesvirus 1 (EHV-1) [33] for borna disease [34,35], both with negative results in the 3 horses.

### 4.2. cDNA Synthesis and Multiplex Tiling PCR

Then, WNV-positive (in nested RT-PCR) RNA samples from washed RBCs were submitted to a cDNA synthesis protocol [36] using a Superscript IV cDNA Synthesis Kit. Then, a multiplex PCR primer scheme was designed (Table S1) to generate complete genomes sequences by means of portable nanopore sequencing, using Primal Scheme (Supplementary Table S1) (http://primal.zibraproject.org accessed on 31 December 2019) [37]. The published WNV genome from Brazil (MH643887) was used to generate a mean 98.4% consensus sequences that formed the target for primer design. The thermocycling conditions involved 40 cycles, and reaction conditions were previously reported in [37].

### 4.3. Library Preparation and Nanopore Sequencing

Amplicons were purified using 1× AMPure XP Beads, and cleaned-up PCR products concentrations were measured using Qubit™ dsDNA HS Assay Kit on a Qubit 3.0 fluorimeter (Thermo Fisher Scientific, Waltham, MA, USA). DNA library preparation was carried out using the Ligation Sequencing Kit and the Native Barcoding Kit (NBD104, Oxford Nanopore Technologies, Oxford, UK) [37]. Purified PCR products pools were pooled together before barcoding reactions (taking in consideration each amplicon pool DNA concentrations), and one barcode was used per sample in order to maximize the number of samples per flow cell. Sequencing library was loaded onto a R9.4 flow cell, and data were collected for up to 6 h, but generally less.

### 4.4. Generation of Consensus Sequences

Raw files were basecalled using Guppy and barcode demultiplexing was performed using qcat. Consensus sequences were generated by de novo assembling using Genome Detective (https://www.genomedetective.com/app/ accessed on 31 December 2019) [38]. New genomes were deposited in the GenBank with accession numbers MW420987, MW420988, and MW420989 (Table 1).

### 4.5. West Nile Virus Typing Tool: Classification Method and Implementation

The classification pipeline we present comprises two components. One for species and sub-species assignment that enables assignment at these levels by BLASTing the query sequences against a set reference sequences [39]. An assignment is made when BLAST reports a result that exceeds the present threshold.

The other component constructs a Neighbor Joining (NJ) phylogenetic tree that is used to make assignments at the lineages and sublineages level. For this component, the query sequence is aligned against a set of reference sequences using the profile alignment option in the ClustalW software [40], such that the query sequence is added to the existing alignment of reference sequences. Following the alignment, a NJ phylogenetic tree with 100 bootstrap replicates is inferred. The tree is constructed using the HKY distance metric with gamma among-site rate variation, as implemented in the PAUP* software (https://paup.phylosolutions.com/ accessed on 31 December 2019) [41]. The query sequence is assigned to a particular genotype if it clusters monophyletically with that genotype clade with bootstrap support >70%. If the bootstrap support is <70%, the genotype is reported to be unassigned (Supplementary Figure S1).

For each of these steps, the earlier discussed reference strains were used with respect to the appropriate typing level (i.e., virus species, lineages, and sublineages). Testing revealed that a BLAST cut-off value of 200 allowed accurate identification of the virus species and WNV using sequence segments >200 base pairs. Note that the species classification procedure is implemented as separate BLAST steps. This enables the tool to efficiently perform large throughput species classification, such as for the classification of shorts sequencing reads. An instance of the web application is publically available on a dedicated server (https://www.genomedetective.com/app/typingtool/wnv/ accessed on 31 December 2019). The web interface on this server accepts up to 2000 whole-genome or partial genome sequences at a time.

### 4.6. Phylogenetic Analysis

The 3 new sequences reported in this study were initially submitted to a genotyping analysis using the new phylogenetic West Nile virus subtyping tool, which is available at https://www.genomedetective.com/app/typingtool/wnv (accessed on 31 December 2019). To put the newly WNV sequences in a global context, we constructed phylogenetic trees to explore the relationship of the sequenced genomes to those of other isolates.

We retrieved 2321 WNV genome sequences with associated lineage date and country of collection from GenBank (Supplementary Figure S2). From this dataset, we generated a subset that included the highly supported (>0.9) clade containing the newly WNV strains obtained in this study plus 29 globally sequences (randomly sampled) from all lineages 1A, 1B, 2, 3, 4, 5, 7, and 8 (Supplementary Table S3). Sequences were aligned using MAFFT [42] and edited using AliView [43]. Those datasets were assessed for the presence of phylogenetic signal by applying the likelihood mapping analysis implemented in the IQ-TREE 1.6.8 software [44]. A maximum likelihood phylogeny was reconstructed using IQ-TREE 1.6.8 software under the HKY+G4 substitution model [44]. We inferred time-scaled trees by using TreeTime [45].

### 4.7. WNV Epidemiological Data

Human reported cases presenting neurological disease compatible with WNV infection collected between November 2015 and early 2020 were obtained from SINAN. We reinforce the nature of the reports as suspected (not confirmed), being officially defined as cases presenting neurological syndromes compatible with WNV infection, registered as suspected occurences of West Nile virus infection (code A923). As such, the spatio-temporal series of suspected cases should only be interpreted as a proxy for the possible spatio-temporal dynamics of WNV infections [46].

### 4.8. Modeling Transmission Potential

To estimate the transmission potential of WNV, we employed the computational approach from Lourenço et al. recently applied in Israel [25] and Portugal [26]. This approach estimates the suitability index P using climatic variables only. The index measures the transmission potential of single adult female mosquitoes (spp. Culex) in the animal reservoir and is thus interpreted as a summary measure of the risk for spill-over into human populations. The theory and practice of estimating the index P for mosquito-borne viruses has been previously described in full by Obolski et al. [27]. The epidemiological priors used were the same as in the original study by Lourenço et al. in Israel, which relate to spp. Culex, WNV, and an average bird species. Climatic data were obtained from Copernicus.eu (https://www.copernicus.eu (accessed on 31 December 2019)); in particular, we used the dataset “essential climate variables for assessment of climate variability from 1979 to present” [47]. This dataset offers climatic variables at a time resolution of 1 month and gridded spatial resolution of 0.25 × 0.25.

## Figures and Tables

**Table 1:** Epidemiological information and sequencing statistics of the three sequenced samples of WNV sampled in Minas Gerais, São Paulo, and Piaui Brazilian states.

ID	Sample	Collection Date	Age	Sex	State	Municipality	Reads	Coverage (%)	Depth of Coverage	Lineage Assignment	Acession Number	Clinical Sign
BC02_07	RBCs	11/07/2018	9 months	F	MG	Sabara	343,743	97.9	6527.6	Lineage 1a	MW420989	Chorioretinitis
BC03_04	RBCs	30/07/2019	13 years-old	M	SP	São Bernardo do Campo	170,980	97.9	3189.7	Lineage 1a	MW420988	Muscle stiffness, tremor retinal and flaccid paralysis
BC05_06	RBCs	21/08/2020	5 years-old	F	PI	Parnaíba	222,516	99.4	4121.4	Lineage 1a	MW420987	Neurological complications

ID = study identifier; RBCs = Red Blood Cells; Collection date = Sample collection date; Municipality = Municipality of residence; State= MG-Minas Gerais; SP = Sao Paulo; PI = Piaui; Sex: M = Male; F = Female; Accession Number = NCBI accession number.

## Data Availability

Newly generated WNV sequences have been deposited in GenBank under accession numbers MW420987, MW420988 and MW420989.

## Supplementary Materials

The following are available online at https://www.mdpi.com/article/10.3390/pathogens10070896/s1, Figure S1: WNV typing tool, Figure S2: Maximum likelihood phylogenetic tree of 2321 WNV complete genomes. Colors indicates different lineages. Highlighted red clade include the WNV viral strain obtained in this study, Table S1: Primer scheme, Table S2: WNV suspected cases reported between 2014–2020 in each Brazilian state, according to SINAN, Table S3: Globally reference WNV sequences from the subset *n* = 29 used in this study.
