# Supplementary material for: West Nile Virus in Brazil
Source: Pathogens. 2021 Jul 15;10(7):896. doi: 10.3390/pathogens10070896 (PMC8308589; doi:10.3390/pathogens10070896)
Supplement: Supplementary file 1 [file pathogens-10-00896-s001.zip › pathogens-1225355-SI.pdf]

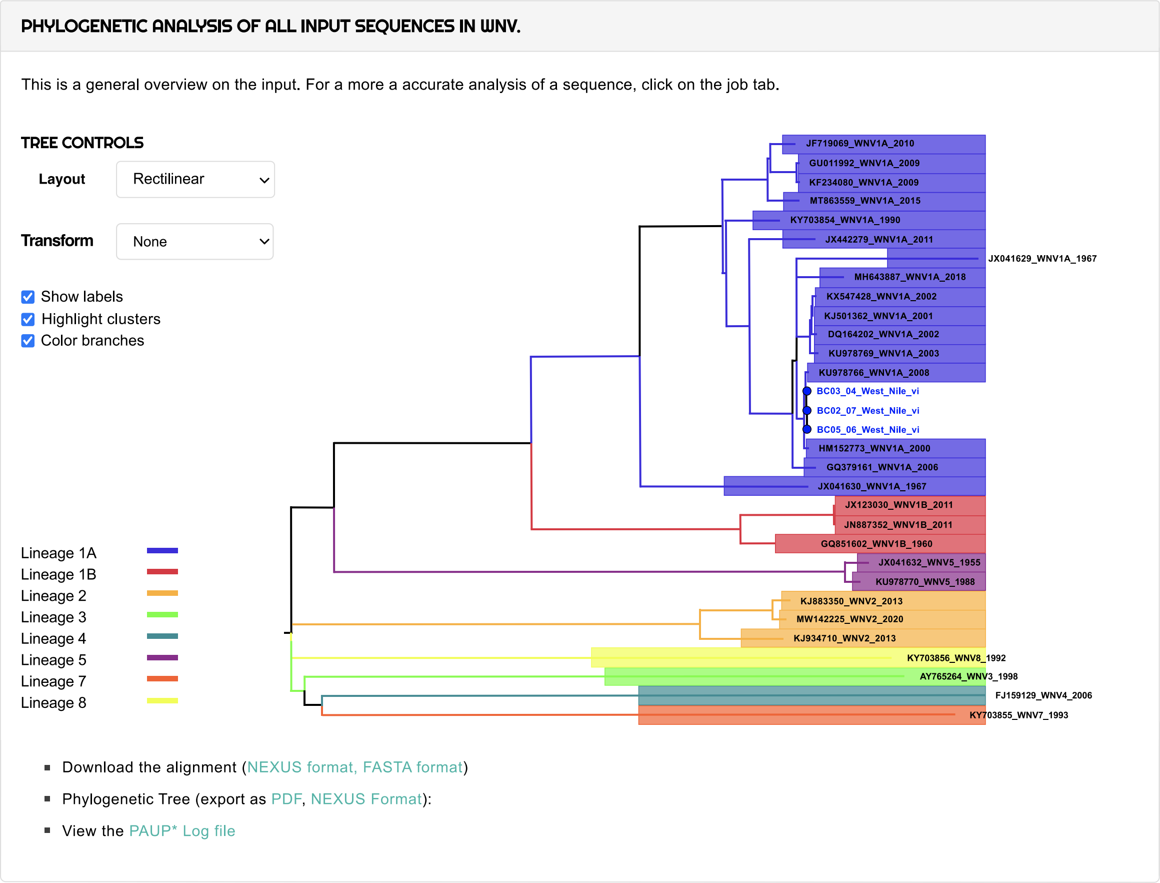

Figure S1. WNV typing tool.

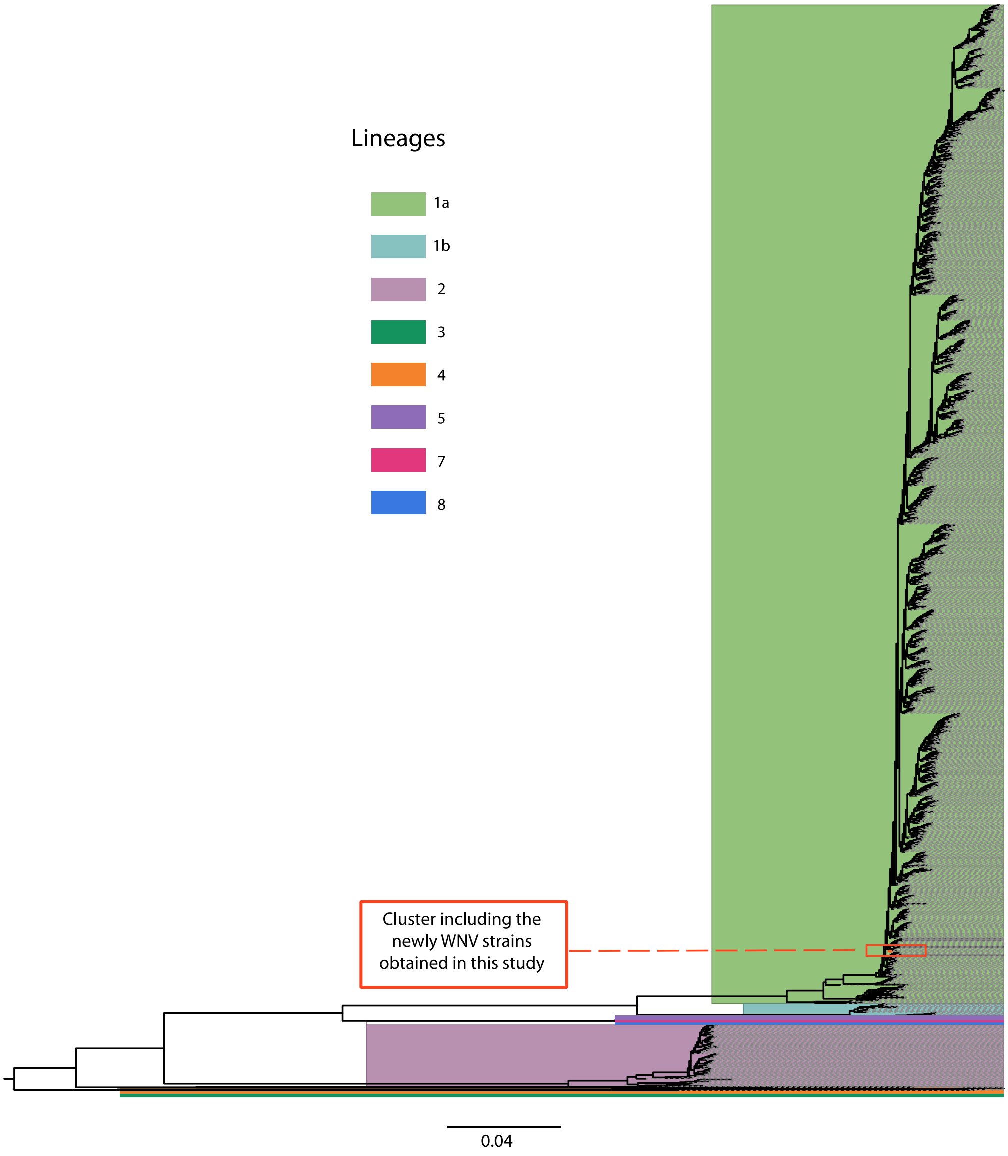

Figure S2. Maximum likelihood phylogenetic tree of 2321 WNV complete genomes. Colours indicates different lineages. Highlighted red clade include the WNV viral strain obtained in this study.

Table S1. Primer scheme.

Primer Name	Sequence
WNVL1a_1_LEFT	GCCTGTGTGAGCTGACAAACTT
WNVL1a_1_RIGHT	TTTGTTTTGAGCTCCGCCGATT
WNVL1a_2_LEFT	GGATCGGTGGAGAGGTGTGAAT
WNVL1a_2_RIGHT	GACTTTGTGCACCAACAGTCGA
WNVL1a_3_LEFT	TGTCAGAGCAATGGATGTGGGA
WNVL1a_3_RIGHT	CTGTTGCTCATTCCAAGGCAGT
WNVL1a_4_LEFT	GTCATTGGTTGGATGCTTGGGA
WNVL1a_4_RIGHT	TGTGTCAATGCTTCCTTTGCCA
WNVL1a_5_LEFT	AATGACAAACGTGCTGACCCAG
WNVL1a_5_RIGHT	CACTCACGATGGACCAAGAACG
WNVL1a_6_LEFT	GGAGAATATGGAGAAGTGACAGTGG
WNVL1a_6_RIGHT	AAAGCCTTTGAACAGACGCCAT
WNVL1a_7_LEFT	TTCAAGCAACACTGTCAAGTTGAC
WNVL1a_7_RIGHT	TTTCCCAATGCTGCTTCCAGAC
WNVL1a_8_LEFT	CCTGATTGAATTGGAACCACCCT
WNVL1a_8_RIGHT	ACGGAGAGGAAGAGCAGAACTC
WNVL1a_9_LEFT	GCATGTCCTGGATAACGCAAGG
WNVL1a_9_RIGHT	AACCACGACACTAAGGTCCACA
WNVL1a_10_LEFT	TCTACGATCAGTTTCCAGACTGGA
WNVL1a_10_RIGHT	GTTGTTCTTGACAGCCGTTCCA
WNVL1a_11_LEFT	AGTGGAGGATTTTGGATTTGGTCT
WNVL1a_11_RIGHT	AGTCAATCTCTACCCGGCCTTC
WNVL1a_12_LEFT	GGCGATGGAATCCTTGAGAGTG
WNVL1a_12_RIGHT	GGCCCAACTGAAAAGGGTCAAT
WNVL1a_13_LEFT	CGGCTGTTGGTATGGTATGGAG
WNVL1a_13_RIGHT	GAAAACAGCCGCCAACATCAAC
WNVL1a_14_LEFT	GGCGACCTTCAAGATACAACCA
WNVL1a_14_RIGHT	GCTAGAGCCAAGCATAGCAGAC
WNVL1a_15_LEFT	GGATACTGCTGTTGATGGTCGG
WNVL1a_15_RIGHT	TCATCAAGCCGCACATCAACTC
WNVL1a_16_LEFT	TTCTGGGAAATCAACAGATATGTGGA
WNVL1a_16_RIGHT	TCAAAGCGGCTCCTTTTGTTGT
WNVL1a_17_LEFT	TCTACAGGATCATGACTCGCGG
WNVL1a_17_RIGHT	CGCTTATGTATGAGCCGTTGGG
WNVL1a_18_LEFT	GACTTTGGACTTCCCCACTGGA
WNVL1a_18_RIGHT	ATTATGTTCTCTGGGCACTGCG
WNVL1a_19_LEFT	ACAGAAGACTGAGAACAGCCGT
WNVL1a_19_RIGHT	TCCAGAGTTCCAAGCTCGATCC
WNVL1a_20_LEFT	TATTCATGACAGCCACCCCACC
WNVL1a_20_RIGHT	GCTGTCACTGCAGATGGTTCTC

WNVL1a_21_LEFT	CTAACTTCAAGGCGAGCAGGGT
WNVL1a_21_RIGHT	TGCAGTCCTCAACAGTTCCAGA
WNVL1a_22_LEFT	TCTACCAACCAGAGCGTGAGAA
WNVL1a_22_RIGHT	CCCAGAACCTCAATGAGCCCTA
WNVL1a_23_LEFT	GAAAGGAAGATTCTGAGGCCGC
WNVL1a_23_RIGHT	CGTTCCTGGAACTTCAGCCATC
WNVL1a_24_LEFT	GTATTCTTCCTCCTCATGCAGCG
WNVL1a_24_RIGHT	CGGCCTCAAGTCCAGAAGAAAC
WNVL1a_25_LEFT	GTTGGCTGGACAAGACCAAGAG
WNVL1a_25_RIGHT	GGAACCATGTAGGCATAGTGGC
WNVL1a_26_LEFT	CTTCGTCGATGTTGGAGTGTCG
WNVL1a_26_RIGHT	CTCCATTCTCCCAAAGCGTCAC
WNVL1a_27_LEFT	GCTGATCTTAGTGTCTCTAGCTGC
WNVL1a_27_RIGHT	CAGTTTTGCTGTGCCCCTAGAG
WNVL1a_28_LEFT	GTACCGCAAAGAGGCCATCATC
WNVL1a_28_RIGHT	TTGACGAGGACTCTCCGATGTC
WNVL1a_29_LEFT	TGGAACATTGTCACCATGAAGAGT
WNVL1a_29_RIGHT	CTTCCTCGTATTGGGGTCCCTT
WNVL1a_30_LEFT	GAGTCGAGCTTCAGGCAATGTG
WNVL1a_30_RIGHT	AGGGAGTAGTGTCAGTCATGGC
WNVL1a_31_LEFT	ACGGCAGTTATGATGTGAAGCC
WNVL1a_31_RIGHT	CTCCTCATCCACCATCTCCCAA
WNVL1a_32_LEFT	GTCAACAGCAATGCAGCTTTGG
WNVL1a_32_RIGHT	GCCAACTTCACGCAGGATGTAA
WNVL1a_33_LEFT	TCGAGGCTCTGGGTTTTCTCAA
WNVL1a_33_RIGHT	TTCCCCTTCCATCATCCTCACC
WNVL1a_34_LEFT	TCCAGAGAAGATCAGAGGGGGA
WNVL1a_34_RIGHT	TGGAACCACCAGTGTTCTTCCA
WNVL1a_35_LEFT	AGAGTGGAAACCGTCAACTGGA
WNVL1a_35_RIGHT	CCAGACCTCCAACATGTCCTCT
WNVL1a_36_LEFT	GTGGCTGCTTCTGTACTTCCAC
WNVL1a_36_RIGHT	TCTACAGTACTGTGTCCTCAACCA
WNVL1a_37_LEFT	GTGGCTATCAACCAAGTCAGAGC
WNVL1a_37_RIGHT	CAACATGTGGGGTCCTTCTTCC
WNVL1a_38_LEFT	GAAGTTGAGTAGACGGTGCTGC
WNVL1a_38_RIGHT	ACGGGGTCTCCACTAACCTCTA

Table S2. WNV suspected cases reported between 2014-2020 in each Brazilian state, according to SINAN.

States	2014	2015	2016	2017	2018	2019	2020
AC	0	0	0	2	0	0	0
AL	2	0	0	0	0	0	0
AM	1	2	0	0	0	0	0
AP	0	0	0	0	0	0	0
BA	0	0	0	0	0	0	0
CE	0	0	0	0	0	1	1
DF	0	0	0	1	0	0	0
ES	0	0	0	1	49	13	0
GO	0	0	0	0	1	2	0
MA	0	1	0	0	1	1	0
MG	2	0	0	0	1	2	0
MS	0	0	0	0	0	0	0
MT	0	0	0	0	2	0	0
PA	0	1	0	0	1	0	0
PB	0	0	0	0	0	0	0
PE	0	0	0	0	0	2	0
PI	10	118	76	2	3	18	5
PR	0	1	0	1	0	1	0
RJ	0	0	0	0	0	0	0
RN	1	0	0	1	4	1	0
RO	0	0	0	0	0	0	0
RR	0	1	1	0	2	0	0
RS	0	0	2	0	0	0	0
SC	0	0	0	1	0	0	0
SE	0	0	0	0	0	0	0
SP	2	5	0	3	8	6	0
TO	0	0	0	0	2	0	0

Table S3. Globally reference WNV sequences from the subset n=29 used in this study.

Acession Number	Collection date	Country	Lineage	Host
MN849176	01/09/2019	USA	1a	Mosquito
JF719069	01/08/2010	Spain	1a	Horse
KY703854	01/01/1990	Senegal	1a	Mosquito
GU011992	01/01/2009	Italy	1a	Human
KF234080	01/01/2009	Italy	1a	Human
MT863559	10/03/2015	France	1a	Horse
JX442279	01/01/2011	China	1a	Mosquito
JX041629	01/01/1967	Azerbaijan	1a	Bird
JX041630	01/01/1967	Azerbaijan	1a	Bird
KX547428	26/09/2002	USA	1a	Mosquito
KJ501362	01/01/2001	USA	1a	Crow
DQ164202	01/01/2002	USA	1a	Human
MH643887	26/04/2018	Brazil	1a	Horse
KU978769	30/05/2003	Mexico	1a	Crow
KU978766	01/07/2009	Colombia	1a	Flamingo
GQ379161	01/02/2006	Argentina	1a	Horse
HM152773	01/01/2000	Israel	1a	Human
JX123030	03/07/2011	Australia	1b	Horse
GQ851602	01/01/1960	Australia	1b	Mosquito
JN887352	01/01/2011	Australia	1b	Horse
KJ883350	05/07/1905	Greece	2	Human
KJ934710	27/08/2013	Romania	2	Tick
MW142225	01/08/2020	Germany	2	Human
AY765264	01/01/1998	Czech Republic	3	Mosquito
FJ159129	01/01/2006	Russia	4	Mosquito
JX041632	01/01/1955	india	5	Mosquito
KU978770	02/12/1988	india	5	Human
KY703855	01/01/1993	Senegal	7	Tick
KY703856	01/01/1992	Senegal	8	Mosquito
